# *Bacillus cereus* pneumonia in an immunocompetent patient: a case report

**DOI:** 10.1186/s40981-017-0096-3

**Published:** 2017-05-08

**Authors:** Yuichiro Shimoyama, Osamu Umegaki, Yukimasa Ooi, Tomoyuki Agui, Noriko Kadono, Toshiaki Minami

**Affiliations:** 1 Department of Anesthesiology, Osaka Medical College, Intensive Care Unit, Osaka Medical College Hospital, 2-7 Daigaku-machi, Takatsuki, Osaka 569-8686 Japan; 2 Department of Internal Medicine, Osaka Medical College, Takatsuki, Japan; 3 Department of Surgery, Osaka Medical College, Intensive Care Unit, Osaka Medical College Hospital, Takatsuki, Japan; 4 Department of Anesthesiology, Osaka Medical College, Takatsuki, Japan

**Keywords:** *Bacillus cereus*, Pneumonia, Immunocompetent patient

## Abstract

**Background:**

*Bacillus cereus* (*B. cereus*) rarely causes lower respiratory tract infections, although most reported cases of *B. cereus* pneumonia are fatal despite intensive antibiotic therapy. We present a case of *B. cereus* pneumonia in an immunocompetent patient.

**Case presentation:**

An 81-year-old woman was transferred from a district general hospital to our hospital for treatment of congestive heart failure. The patient presented with a nonproductive cough, dyspnea, edema in both lower extremities, orthopnea, fever, and occult blood in the stool. A chest radiograph indicated bilateral pleural effusion and pulmonary congestion. After diuretic therapy and chest drainage, bilateral pleural effusion and pulmonary congestion improved. On day 2, she experienced severe respiratory distress. *B. cereus* was isolated from two blood sample cultures. On day 4, her condition had progressed to severe respiratory distress (PaO_2_/FiO_2_ ratio = 108). A chest radiograph and computed tomography indicated extensive bilateral infiltrates. She was transferred to the intensive care unit and was intubated. *B. cereus* was also isolated from five blood sample cultures at that time. After isolating *B. cereus*, we switched antibiotics to a combination of imipenem and levofloxacin, which were effective. She had no history of immunodeficiency, surgery, ill close contacts, risk factors for HIV or tuberculosis, recent central venous catheter insertion, or anthrax vaccination. She improved and was discharged from the intensive care unit after several days.

**Conclusion:**

This is a rare case of *B. cereus* pneumonia in an immunocompetent patient, who subsequently recovered. *Bacillus* should be considered as a potential pathogen when immunocompetent patients develop severe pneumonia.

## Background

*Bacillus cereus* (*B. cereus*) is a Gram-positive, aerobic to facultative, spore-forming rod that is widely distributed in the environment [1]. *B cereus* is occasionally associated with food-borne illness, its presence in cultures is often considered a contaminant, and it is typically not further characterized beyond a descriptive identification and may not be reported at all [2]. However, severe hematogenous infections caused by *B. cereus* have been reported, especially in drug addicts, premature neonates, and patients with severe underlying diseases or compromised immunity [3]. *B. cereus* rarely causes lower respiratory tract infections, although most reported cases of *B. cereus* pneumonia are fatal despite intensive antibiotic therapy [3]. Here we present a case of *B. cereus* pneumonia in an immunocompetent patient.

## Case presentation

**Fig. 1:**
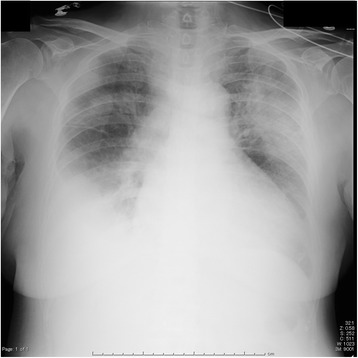
Chest radiograph indicated bilateral pleural effusion and pulmonary congestion

**Fig. 2:**
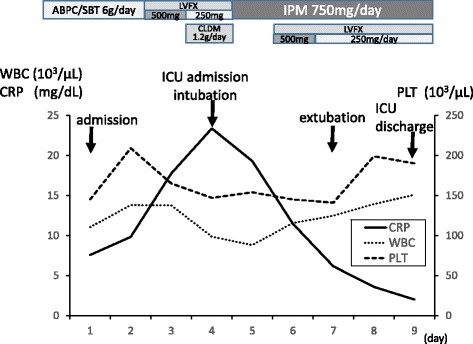
Time course of laboratory data and antibiotic treatment

**Fig. 3:**
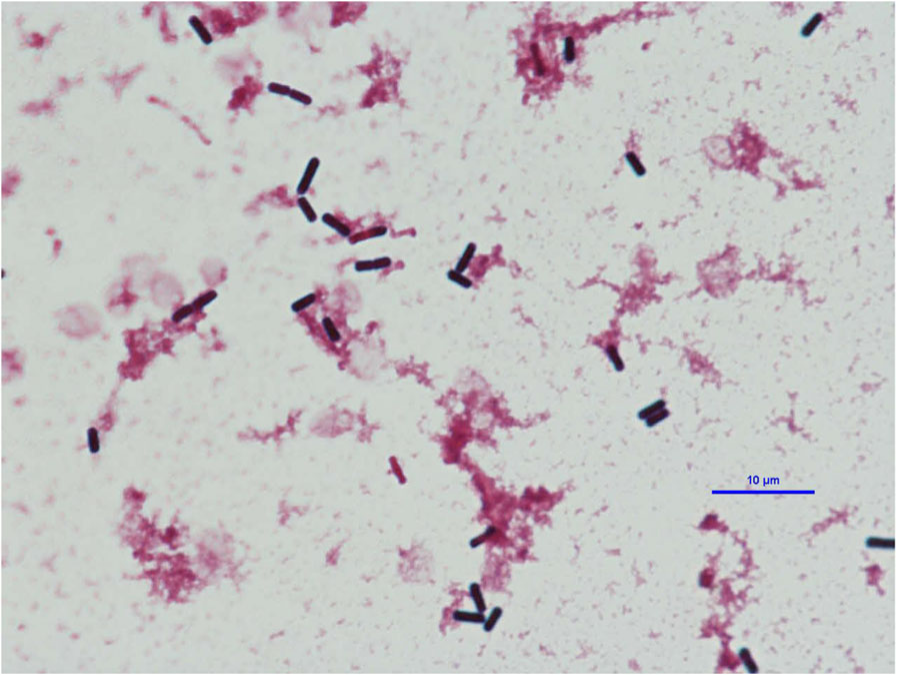
*Bacillus cereus* was isolated from two blood sample cultures collected at admission

**Fig. 4:**
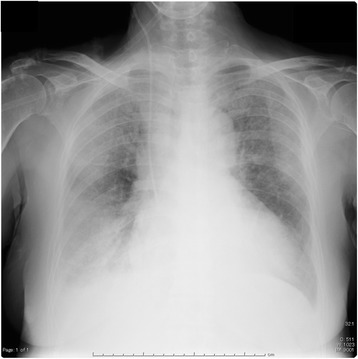
Chest radiograph indicated extensive bilateral infiltrates

**Fig. 5:**
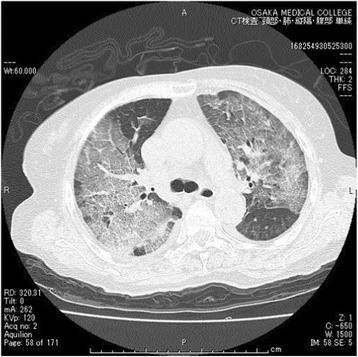
Computed tomography indicated extensive bilateral infiltrates

An 81-year-old woman (160 cm, 60 kg) was transferred from a district general hospital to our hospital for treatment of congestive heart failure. At admission, the patient presented with a nonproductive cough, dyspnea, edema in both lower extremities, orthopnea, fever, and occult blood in the stool, all of which began to about 10 days prior to admission. Past medical history included atrial fibrillation (AF). She had a no drinking and smoking history, and activities of daily living were in the normal range. Her temperature was 37.3 °C, pulse rate was 133 beats per min with AF, respiratory rate was 24 breaths per min, and blood pressure was 115/58 mmHg. Laboratory findings were as follows: WBC count, 11,040 cells/mm^3^ without a left shift; hematocrit, 34.7%; platelets, 145,000/mm^3^; creatinine, 0.8 mg/dL; alanine aminotransferase, 69 U/mL; aspartate aminotransferase, 56 IU/mL; total bilirubin, 1.4 mg/dL; and respiratory alkalosis. A chest radiograph indicated bilateral pleural effusion and pulmonary congestion (Fig. 1). We performed transthoracic echocardiography (TTE). TTE showed an ejection fraction of 50%; verrucas were not detected. Intravenous ampicillin/sulbactam (6 g/day) therapy was initiated to treat a potential bacterial infection (Fig. 2). After diuretic therapy and chest drainage, bilateral pleural effusion and pulmonary congestion improved. On day 2, she experienced severe respiratory distress, with diminished breath sounds, bilateral pulmonary crackles, and an oxygen saturation of 98% while receiving 100% oxygen through a nonrebreather 4-L mask. *B. cereus* was isolated from two blood sample cultures (arterial blood and venous blood) collected at admission (Fig. 3), and intravenous levofloxacin (250–500 mg/day) therapy was initiated instead of ampicillin/sulbactam therapy (Fig. 2). No pathogenic organisms were identified by sputum and pleural effusion of chest drain cultures. On day 3, clindamycin (1200 mg/day) was combined with levofloxacin (Fig. 2). On day 4, her condition progressed to severe respiratory distress (PaO_2_/FiO_2_ ratio = 108). A chest radiograph (Fig. 4) and computed tomography (CT) (Fig. 5) indicated extensive bilateral infiltrates. Her temperature was 37.5 °C, pulse rate was 139 beats per min with AF, blood pressure was 78/47 mmHg, and oxygen saturation was 93% while receiving 100% oxygen by a rebreather 15-L mask. She was transferred to the intensive care unit (ICU) and intubated. She had pulmonary coarse crackles, and moderate amounts of frothy pale pink respiratory secretions were collected from the endotracheal tube. Dopamine, dobutamine, and noradrenaline were administered for hypotension. Laboratory findings were as follows: WBC count, 9860 cells/mm^3^ without a left shift; hematocrit, 37.0%; platelets, 147,000/mm^3^; creatinine, 1.47 mg/dL; alanine aminotransferase, 98 IU/mL; aspartate aminotransferase, 54 IU/mL; total bilirubin, 1.5 mg/dL; and respiratory alkalosis. The patient was unable to mount an adequate leukocyte count in light of her clinical condition. *B. cereus* was isolated from five blood sample cultures (four arterial blood cultures and one venous blood culture), but stool cultures were negative at that time.

On day 4, intravenous imipenem (750 mg/day) therapy was initiated instead of levofloxacin and clindamycin therapy (Fig. 2). On day 5, levofloxacin (250–500 mg) was combined with imipenem (Fig. 2). She had no history of immunodeficiency, surgery, ill close contacts, risk factors for HIV or tuberculosis, recent central venous catheter insertion, or anthrax vaccination. On day 7, a chest radiograph and CT indicated a reduction in the size of bilateral infiltrates, suggesting that respiratory therapy and antibiotic therapy were effective. The patient was extubated and, on day 9, discharged from the ICU. No further remarkable changes were noted, and she was discharged from the hospital.

### Discussion

The present case was unusual, given that the patient was not immunocompromised but succumbed to lung infiltrates associated with *B. cereus. B. cereus* is a well-known pathogen in food poisoning. It causes toxin-mediated, self-limited illness characterized by emetic or diarrheal syndromes [4, 5]. It is also known to cause bacteremia [6], endocarditis [7], meningitis [8], and pneumonia [2, 3, 9–20].

Previously reported cases of *B. cereus* pneumonia in adults are rare and usually associated with significant risk factors which are summarized in Table 1. Immunocompromised patients with *B. cereus* pneumonia frequently have septicemia and fatal illness. Most previously reported cases of infection occurred in patients with hematological disorders or alcohol abuse. However, four lethal cases of *B. cereus* pneumonia in immunocompetent welders and metalworkers have been reported [2, 10]. Our patient had no history of immunodeficiency, surgery, ill close contacts, risk factors for HIV or tuberculosis, recent central venous catheter insertion, or anthrax vaccination. It is interesting that our patient, who had none of the known risk factors for severe respiratory illness, survived the episode. However, the patient’s age (81 years) may have been a contributing factor. As age advances, the immune system undergoes profound remodeling and decline. This immune senescence predisposes older adults to a higher risk of acute viral and bacterial infections [21]. Compared to previously reported episodes of *B. cereus* pneumonia in adults (Table 1), our patient was much older and thus may have been more susceptible to *B. cereus* infection*.*

**Table 1:** Reported episodes of *B. cereus* pneumonia in adults

Patient	Age	Risk factor	Outcome	Reference
1	Not available	None	Died	20
2	52	Leukemia	Died	19
3	63	Leukemia	Died	18
4	29	Leukemia	Recovered	17
5	60	Alcohol abuse	Recovered	16
6	18	Alcohol abuse	Recovered	15
7	54	Leukemia	Recovered	14
8	21	Bronchiectasis	Recovered	13
9	46	None (welder)	Died	2
10	41	None (welder)	Died	2
11	52	Aplastic anemia	Died	12
12	37	Leukemia	Died	11
13	39	None (metal worker)	Died	10
14	56	None (metal worker)	Died	10
15	60	Leukemia	Died	9
16	43	Nephrotic syndrome	Recovered	3

Non-anthracis *Bacillus* species might be dismissed as contaminants when isolated from clinical samples. Therefore, detection of the microorganism in multiple samples is usually necessary to make a definitive diagnosis of *B. cereus* pneumonia [3].

In the present case, the pathogenic role of *B. cereus* in pneumonia was confirmed by seven different specimens of blood that were sampled aseptically. However, we did not perform a bronchoscopic assessment of lung infiltrates, and *B. cereus* was not confirmed from bronchial lavage fluid and transbronchial lung biopsy specimens. There were no episodes of infectious disease other than severe pneumonia during hospitalization. Respiratory symptoms such as cough and dyspnea, and gastrointestinal symptoms (occult blood in the stool) were present. Therefore, in the present case, *B. cereus* pulmonary infection may have resulted from transient bacteremia from a gastrointestinal infection. Indeed, we had suspected as such at a relatively early stage (on day 2). *B. cereus* produces β-lactamase and is therefore resistant to penicillin and cephalosporins [1]. *B. cereus* is usually susceptible to clindamycin, vancomycin, fluoroquinolones, carbapenems, and aminoglycosides. After isolating *B. cereus*, we switched antibiotics to a combination of imipenem and levofloxacin, which were effective.

## Conclusions

In summary, we report a rare case of *B. cereus* pneumonia in an immunocompetent patient, who subsequently recovered. Healthcare providers should consider *Bacillus* species as a potential pathogen when immunocompetent patients develop severe pneumonia.

## Abbreviations

AF: Atrial fibrillation
*Bacillus cereus*: *B. cereus*
CT: Computed tomography
ICU: Intensive care unit
TTE: Transthoracic echocardiography
